# Annual growth cycle observation, hybridization and forcing culture for improving the ornamental application of *Paeonia lactiflora* Pall. in the low-latitude regions

**DOI:** 10.1371/journal.pone.0218164

**Published:** 2019-06-13

**Authors:** Jiaping Zhang, Dong Zhang, Jianfen Wei, Xiaohua Shi, Huaqiao Ding, Shuai Qiu, Juan Guo, Danqin Li, Kaiyuan Zhu, David P. Horvath, Yiping Xia

**Affiliations:** 1 Physiology and Molecular Biology Laboratory of Ornamental Plants, Institute of Landscape Architecture, College of Agriculture & Biotechnology, Zhejiang University, Hangzhou, Zhejiang Province, China; 2 Research & Development Center, Hangzhou Landscaping Incorporated, Hangzhou, Zhejiang Province, China; 3 Research & Development Centre of Flower, Zhejiang Academy of Agricultural Sciences, Hangzhou, Zhejiang Province, China; 4 Sunflower and Plant Biology Research, Red River Valley Agricultural Research Center, Agricultural Research Service, United States Department of Agriculture, Fargo, North Dakota, United States of America; Institute for Horticultural Plants, China Agricultural University, CHINA

## Abstract

Expanding the southern range of herbaceous peony (*Paeonia lactiflora* Pall.) is a meaningful and worthwhile horticultural endeavor in the Northern Hemisphere. However, high temperatures in winter seriously hinder the bud dormancy release and flowering of peony in the more southern areas of subtropical and tropical regions. Resource introduction and hybridization can contribute to creating new cultivars with high adaptability in a warmer winter climate. In this study, three representative cultivars of *P*. *lactiflora* were screened for flowering capabilities and their annual growth cycles were observed to provide information needed for hybridization. Among these three cultivars, ‘Hang Baishao’ is the best adapted cultivar for southern growing regions and is unique in its ability to thrive in southern areas of N 30°00’. Pollen viability of ‘Hang Baishao’ was 55.60% based on five measuring methods, which makes it an excellent male parent in hybridization. Hybrid plants among these three cultivars grew well, but all of their flower buds aborted. Additionally, the ability of three growth regulators that advance the flowering of ‘Hang Baishao’ to promote an indoor cultivation strategy for improving peony application as a potted or cut-flower plant was tested. 5-azacytidine could impact the growth of ‘Hang Baishao’ and induce dwarfism and small flowers but not advance the flowering time. Gibberellin A_3_ promoted the sprouting and growth significantly, but all plants eventually withered. Chilling at 0–4°C for four weeks and irrigation with 300 mg/L humic acid was the optimal combination used to hasten flowering and ensure flowering quality simultaneously. These results can lay the foundation for future studies on the chilling requirement trait, bud dormancy release and key functional gene exploration of herbaceous peony. Additionally, this study can also provide guidance for expanding the range of economically important plants with the winter dormancy trait to the low-latitude regions.

## Introduction

Artificially extending the planting range of economically important plants is an important subject in horticultural research[1]. Unsuitable climatic conditions, such as high winter temperatures in a new planting region can be problematic and may prevent or reduce growth and flowering due to the loss of vernalization and/or dormancy breaking conditions [2–4]. With the trend of global warming, research on the detrimental impact of high winter temperatures has been of significantly greater focus compared with research on high summer temperatures in the recent years[5]. Warm winters lead to a serious deficiency of chilling accumulation, incomplete dormancy release, weak growth or low yield for deciduous fruit trees, tea trees, ornamentals and other economically important plants with winter dormancy traits[1,6–8]. Taking the herbaceous peony (*Paeonia lactiflora* Pall.), for example, this is a valuable ornamental flower that is widely cultivated in European, Asian and North American regions in the Northern Hemisphere with a temperate or cool climate [9,10]. Partial cultivars of herbaceous peony can grow in the northern subtropical regions; however, most cultivars cannot be cultivated or grown well in the low-latitude areas, such as central and southern subtropics and tropics. Although a few cultivars can be planted in these regions, they bloom very poorly[1,11,12].

Therefore, promoting peony cultivation and ornamental utilization in the low-latitude regions is desirable but problematic[1,13]. Peony is a perennial geophyte and its underground renewal buds must go through a crucial chilling period to break dormancy and reinitiate sprouting, growth and subsequent blooming[14–17]. Warm winters causes serious chilling deficiency, abnormal bud dormancy release and sprouting, reduced or absent flowers, rapid crown degradation and eventual death, which greatly impedes the intensive production and wide utilization of herbaceous peony in the central subtropical regions, such as Zhejiang Province (N 27°12’-N 31°31’, E 118°- E 123°) in southern regions of the Yangzi River of China[5,12,13]. Resource introduction and crossbreeding for the creation of new cultivars with the special trait of a low chilling requirement will promote the cultivation of peony in subtropical and tropical gardens and parks[13,14,18]. Additionally, improving glasshouse cultivation and regulating flowering will facilitate the year-round production and indoor application of herbaceous peony as a potted or cut-flower. Forcing culture, one type of flowering regulation, can allow for pollen collection in advance for cultivars with late flowering periods and allow use of these normally late-flowering cultivars as male parents in hybridization [10,19,20]. These two aspects will simultaneously increase outdoor and indoor planting and utilization of peony in the low-latitude regions.

**Fig 1:**
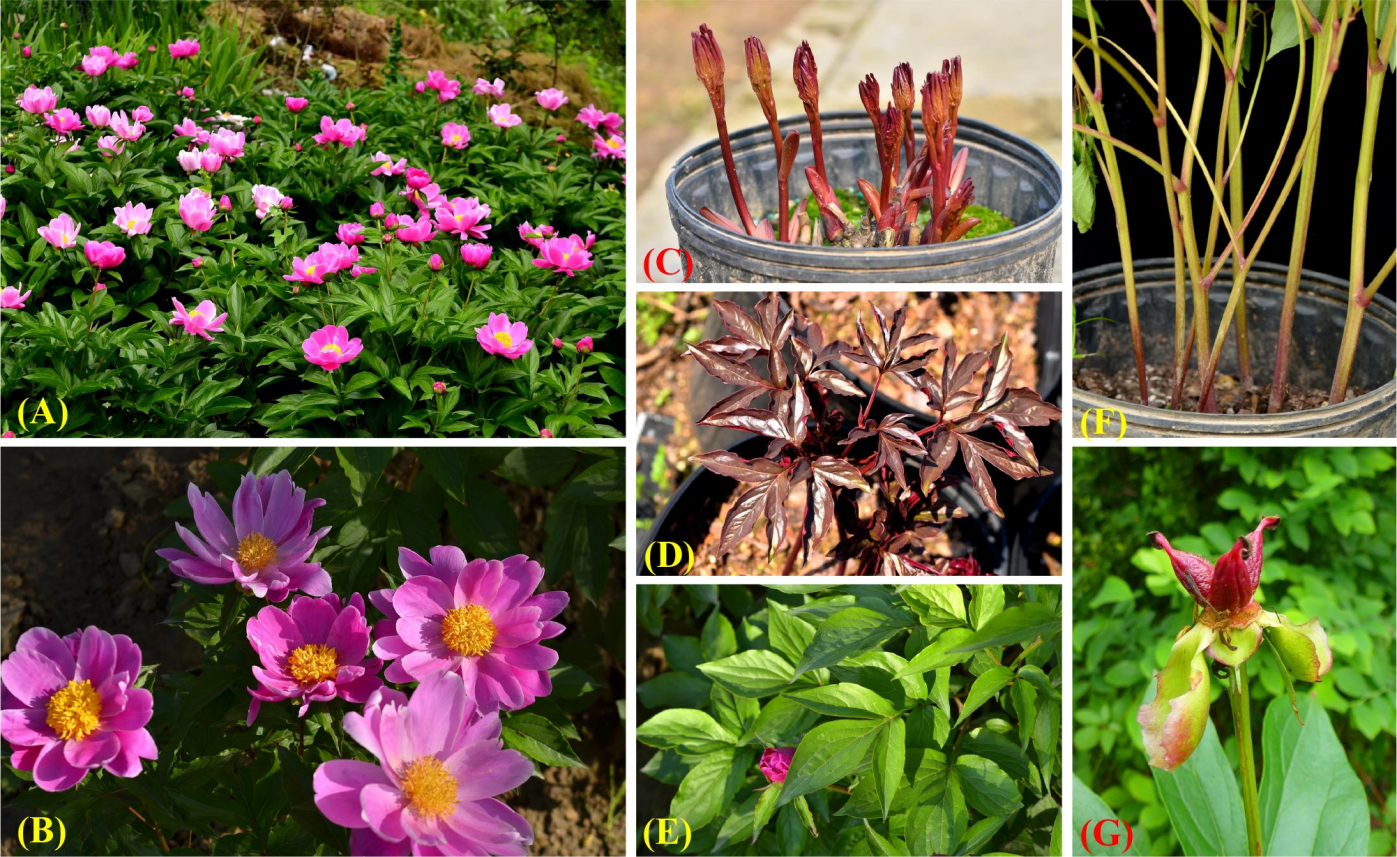
Planting and ornamental value of *P*. *lactiflora* ‘Hang Baishao’, which is the southernmost cultivated herbaceous peony in eastern China. (A)—(B): landscapes with plants in full bloom; (C): bright purple-red tender buds and stems; (D)—(E): tender and mature leaves; (F): mature stems; (G) charming red mature follicles.

A unique herbaceous peony, *P*. *lactiflora* Pall. ‘Hang Baishao’, has been planted in Zhejiang Province as a Chinese medicinal crop for hundreds of years[1,11]. It is also valued for its beautiful single-flowers, charming purple tender buds, stems and leaves, and red mature follicles (Fig 1)[12,21]. Compared to many other *P*. *lactiflora* cultivars introduced from northern China, ‘Hang Baishao’ can grow and bloom vigorously under relatively warm winters and hot summers in Zhejiang[1]. ‘Hang Baishao’ is unique that it is the only cultivar that can be intensively planted and widely used in the Chinese low altitude regions (the southern region of N 30°00’), and has special values on landscape application, crossbreeding and scientific research.

**Fig 2:**
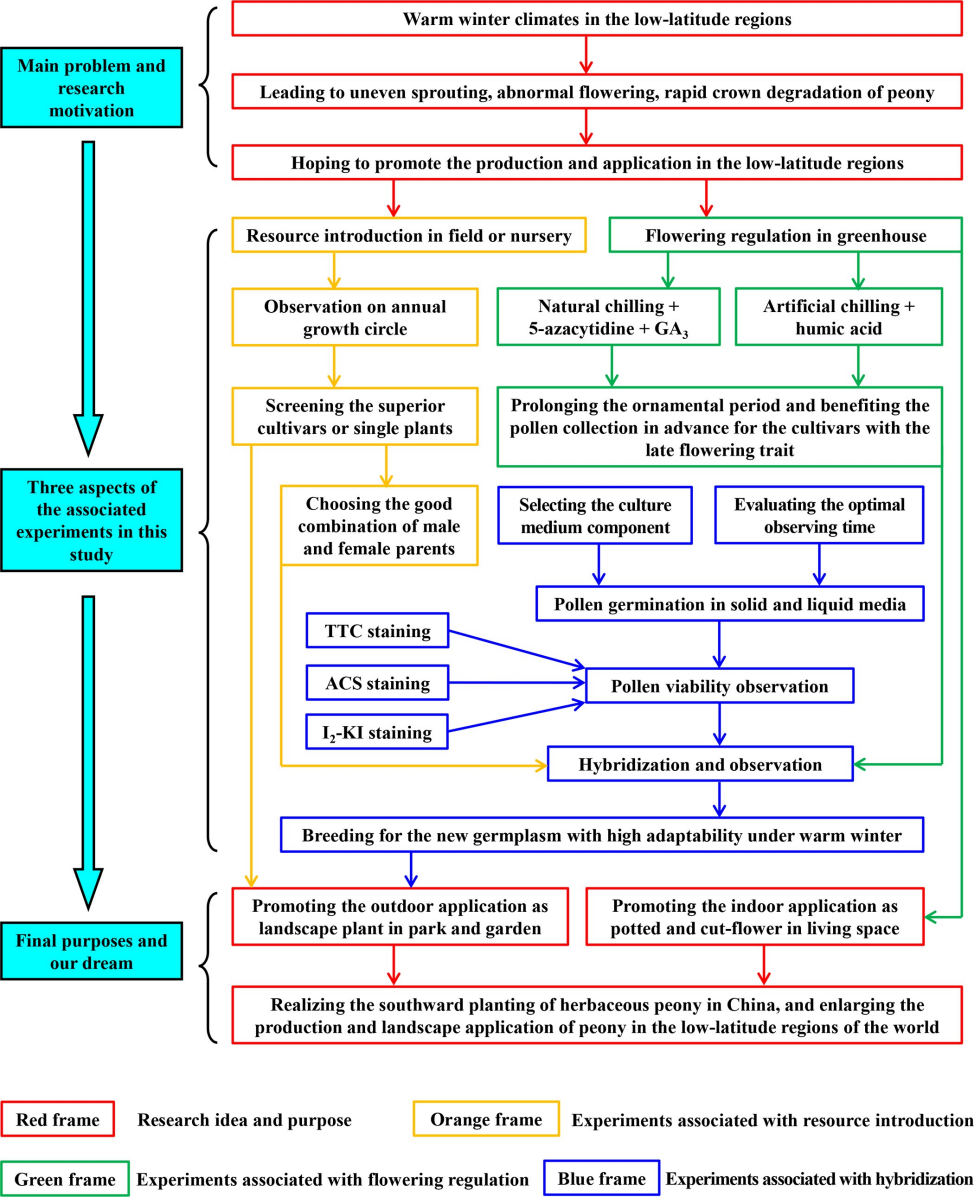
Research motivation, framework and final purpose of this study.

Based on above, in this study, we used ‘Hang Baishao’ and two other representative northern peonies as the specific materials and observed and compared their annual growth cycles and comprehensive performances. Hybridization among these three cultivars was performed in order to breed new cultivars with both high ornamental value and low chilling requirements. We also explored the best strategy for the forcing culture of ‘Hang Baishao’ by using three exogenous reagents and two types of chilling treatment (Fig 2). The results of this study can contribute to the popularity and utilization of herbaceous peony in more southern regions of China and other countries in the Northern Hemisphere[1,11,12,22]. This study can also be seen as an example of how to systematically promote the development and use of important economic crops to the low-latitude regions in the field and greenhouse simultaneously.

## Materials and methods

### Plant materials and observation of annual growth cycles

**Fig 3:**
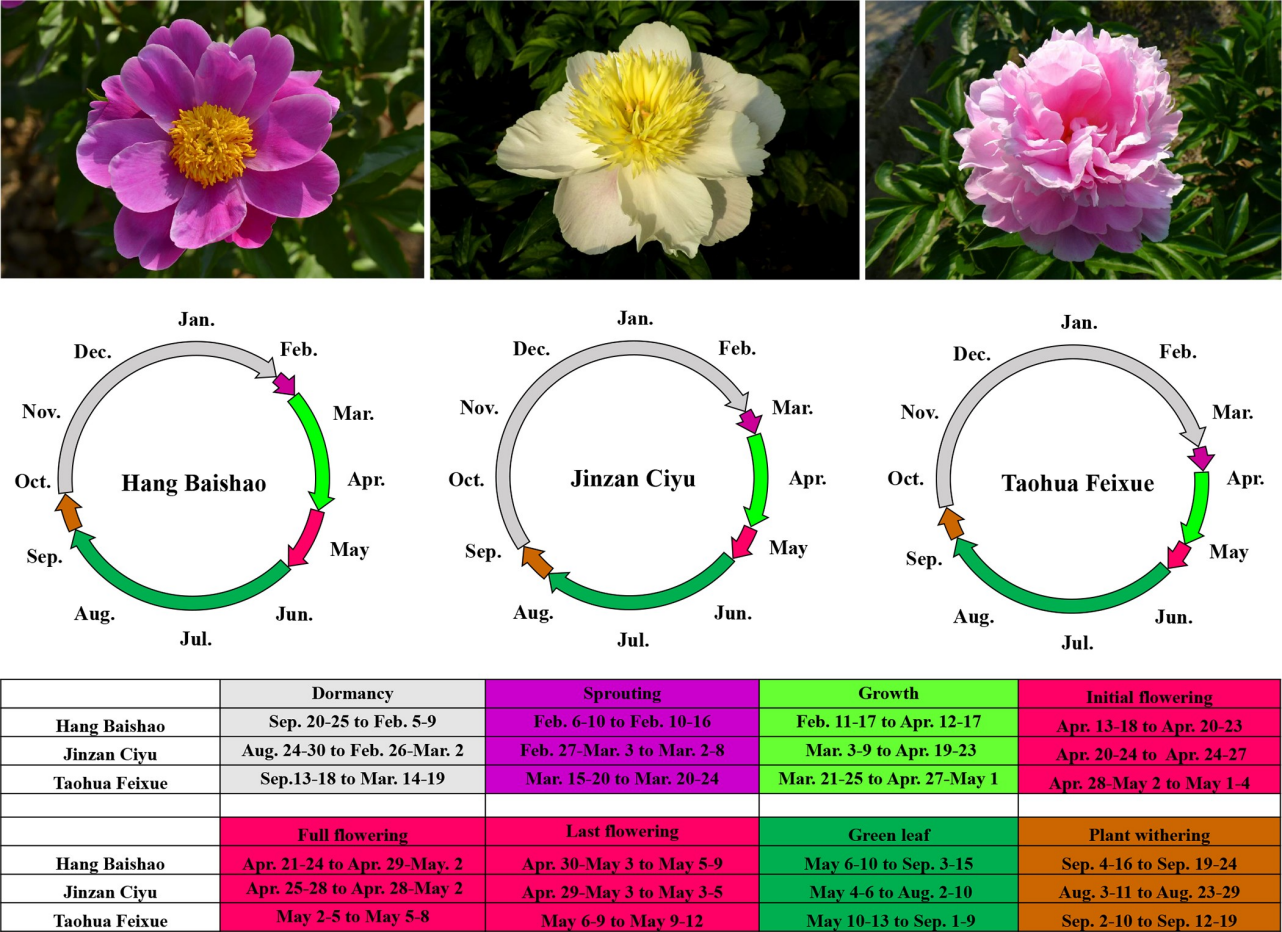
Flowers and comparison of the annual growth cycles among three cultivars.

Research motivation, framework and three main aspects of the associated experiments in this study are shown in detail in Fig 2. Based on a comprehensive evaluation of the growth and flowering performances of dozens of herbaceous peony cultivars introduced from northern and southern China (unpublished data), three typical cultivars were selected as the main materials in this study, which have significantly different traits of dormancy, sprouting, growth, flowering and senescence (Table 1, Fig 3).

**Table 1:** Main information of three representative cultivars with typical early, intermediate and late sprouting and flowering.

Cultivar name	Provenance	Longitude and latitude	Flower form	Ploidy	Flowering
Hang Baishao	Jinhua, Zhejiang Province	E 120°17’-120°47’, N 28°49’-29°19’	Single form	Diploid	Early
Jinzan Ciyu	Heze, Shandong Province	E 114°48’ -116°24’, N 34°39’ -35°52’	Anemone form		Intermediate
Taohua Feixue			Crown form		Late

The underground crowns of ‘Hang Baishao’, ‘Jinzan Ciyu’ and ‘Taohua Feixue’ (Table 1) were cultivated under natural light, normal watering and fertilization in the Perennial Flower Resources Garden of Zhejiang University in Hangzhou (E 118°21’-120°30’, N 29°11’-30°33’), Zhejiang Province. Culture medium consisted of garden soil:peat:perlite at 7:2:1 by volume. Annual growth cycles of the three cultivars were observed and compared from 2011 or 2012 to 2017 to collect enough information needed for subsequent hybridization and forcing culture [23]. A randomized complete block experimental design with three blocks and three technical replicates per block was used. The indices related to growth, such as plant height and width, and stem number and diameter were recorded during the full-bloom stage every year. Average blooming durations were calculated as the number of days from flower opening to withering. Flower opening was considered to occur when stamens could be visually detected, and withering was defined as the initiation of natural petal abscission.

### Pollen viability and hybridization between southern and northern peonies

#### Measurements of pollen viability using five methods

Pollen viability was measured to provide information for subsequent pollination. Two germination methods were used: one using solid culture medium (GS method) and another using liquid culture medium (GL method).

For the GS method, pollen grains were inoculated on solid medium and then incubated at 25°C and 80% relative humidity. First, two key problems in the culture of pollen germination were explored with media 2 and 3, including the appropriate period and optimal time for observing pollen germination, and the necessity of CaCl_2_ application (Table 2). The germination rates were measured continuously at intervals of 0.5 h (up to 6.0 h) under the Splan 10PL (0.30, 160/0.17) lens of an Olympus BH-2 light microscope (Shanghai BM optical instruments manufacture company LTD, China). Second, media with different agar concentrations (media 1, 2 and 4 in Table 2) were prepared, and pollen germination was observed to identify the best medium components according to the previously acquired optimal observation period. Germination of pollen was defined as occurring when the length of the elongated tube was equal to or longer than the diameter of the pollen grain[24–28].

**Table 2:** Components and pollen germination rates observed three hours after inoculation in different culture media.

Number	Culture medium	Pollen germination rate(%)	Pollen submergence time after inoculation (h)
1	Sucrose 100 g/L+ Boric acid 20 mg/L+ Agar powder 5 g/L	57.57 ± 0.56 b^**Z**^	2.46 ± 0.05 b
2	Sucrose 100 g/L+ Boric acid 20 mg/L+ Agar powder 8 g/L	63.30 ± 0.77 a	4.03 ±0.04 a
3	Sucrose 100 g/L+ Boric acid 20 mg/L+ Agar powder 8 g/L + CaCl_2_ 25 mg/L	21.06± 0.46 c	
4	Sucrose 100 g/L+ Boric acid 20 mg/L+ Agar powder 10 g/L	62.57 ± 0.24 a	4.08 ± 0.05 a

^**Z**^The symbols of a, b, c and d in this table indicate the statistical significance of the differences, which are the same meaning with those in the subsequent partial tables and “S# Tables” of this article.

Additionally, for the GL method, three staining methods for determining pollen viability were tested, including the 2,3,5-triphenyl tetrazolium chloride staining method (TTC method, 0.5%), aceto-carmine staining method (ACS method, 1%), and I_2_-KI staining method (IKS method, 2%). Solutions of TTC, ACS and IKS were prepared and dropped onto glass slides to cover the pollen grains; those that stained completely were defined as alive[29–32].

#### Hybridization between the southern ‘Hang Baishao’ and northern peonies

Hybridizations between ‘Hang Baishao’, ‘Jinzan Ciyu’ and ‘Taohua Feixue’ were made continuously for four years from spring of 2013 to 2016. According to the observation of annual growth cycle for the three cultivars, ‘Hang Baishao’ was the best performing and had the highest adaptability to the warm winters in Hangzhou, and it also had the earliest flowering development with high pollen quantity and activity. Therefore, we selected it as the male parent in all crosses. ‘Jinzan Ciyu’ and ‘Taohua Feixue’ were chosen as the female parents because of their distinct stigmas and pollen deficiency. Anthers of ‘Hang Baishao’ were collected in the full flowering on sunny and windless days and dried naturally by scattering the pollen on a filter paper in glass dishes. Two northern cultivars were emasculated and then pollinated artificially by dipping the stigma into dried pollen or by using a cotton swab to place the pollen onto the stigma. The pollinated whole flowers were immediately covered with waxed paper bags and labeled [23,33,34]. Hybrid seeds were collected in mid-July when they were fully ripe (follicles became red) and immediately sown in a seedbed or in one-gallon pots (five seeds per pot, filled with 7 parts garden soil:2 parts peat:1 part perlite by volume). Seed number, weight, diameter and germination rate, and plant heights of one- and two-year-old seedlings were recorded to evaluate the performance of the hybrid offspring. Height of the hybrid plants with flower buds and the percentage of aborted flowers were also measured. Two northern cultivars had no stamens and pollen, and hybrid plants had no normal flowers, thus, reciprocal and backcrosses were not attempted.

### Forcing culture for ‘Hang Baishao’ under three exogenous reagents and two chilling treatments

#### Experiment Ⅰ: Natural low temperature, 5-azacytidine and gibberellin A3

‘Hang Baishao’ has strong adaptability under the warm winter climate in Hangzhou, typical early flowering and high potential for advancing the flowering period according to the results associated with the annual growth cycle in this study and comprehensive evaluation of dozens of cultivars (unpublished data). Thus, in this study, ‘Hang Baishao’ was selected as a material for investigating the forcing culture of peony south of the Yangtze River.

Outdoor potted crowns of ‘Hang Baishao’ were transferred from nursery into a glasshouse (15–25°C, natural light levels and normal watering) at 9:00 a.m. on four transfer dates at an interval of four or five weeks from November 26, 2012 to February 25, 2013 (Table 3). Subgroups of the potted crowns for each transfer date were assigned as follows: in the second subgroup (T. *-2 in Table 3), the dormant buds on crowns were painted by a brush pen immersed in 5-azacytidine solution (SIGMA, USA; 40 mg/L; NLT+5-azac in Table 3); in the third subgroup (T. *-3), the potted crowns were irrigated with gibberellin A3 (GA_3_) (SIGMA, USA; 300 mg/L, 150 mL per pot; NLT+GA_3_ in Table 3); and in the fourth subgroup (T. *-4), buds were painted with 5-azacytidine, and crowns were irrigated by GA_3_ simultaneously (NLT+5-azac+GA_3_ in Table 3). These three treatments were performed three times in November of 2012 before the first transfer date (seven pots per replicate and three replicates per treatment).

**Table 3:** Details of 20 treatments for the forcing culture of ‘Hang Baishao’ under naturally low temperature, 5-azacytidine and/or GA_3_.

Treatment detail	Nov. 26, 2012^Z^ (0 h^Y^)	Dec. 24, 2012 (250 h)	Jan. 21, 2013 (738 h)	Feb. 25, 2013 (1,170 h)	Always outdoor
Only NLT ^**X**^	T. ^**W**^1–1	T. 2–1	T. 3–1	T. 4–1	T. 5–1
NLT+5-azac^**V**^	T. 1–2	T. 2–2	T. 3–2	T. 4–2	T. 5–2
NLT+GA_3_	T. 1–3	T. 2–3	T. 3–3	T. 4–3	T. 5–3
NLT+5-azac+GA_3_	T. 1–4	T. 2–4	T. 3–4	T. 4–4	T. 5–4

^**Z**^The date of transferring into glasshouse.

^**Y**^Number of hours when the treatment temperatures were between 0.0 to 7.2°C in this transfer-date, i.e. chilling hours, which can reflect the chilling accumulation of the treated plants, and had already been elaborated and analyzed in previous publication[1]

^**X**^NLT: **n**atural **l**ow **t**emperature.

^**W**^T.: **tre**atment.

^**V**^5-azac: **5-azac**ytidine.

#### Experiment Ⅱ: Artificial low temperature and humic acid

The potted crowns were treated in refrigerated storage (0–4°C, cleaned and sterilized, without light and water) at 9:00 a.m. on Nov. 29, 2013, then transferred into a glasshouse on six transfer dates with one-week intervals (Table 4, chilling treatment for one to five weeks in refrigerated storage). The plants of “T. 6-*” in Table 4, which received no artificial chilling, were directly transferred into a greenhouse on Nov. 29. The potted crowns were irrigated with humic acid (HA) (Ziming Organic Agricultural Chemistry, China) at concentrations of 0, 300, 600 and 900 mg/L (250 mL/pot) twice a week until flowering in glasshouse (Table 4, three replicates and three pots per replicate).

**Table 4:** Detail of 24 treatments for the forcing culture of ‘Hang Baishao’ under artificial low temperature and HA treatment.

Treatment detail	Nov. 29, 2013^Z^ (0 h^Y^)	Dec. 6, 2013 (168 h)	Dec. 13, 2013 (336 h)	Dec. 20, 2013 (504 h)	Dec. 27, 2013 (672 h)	Jan. 3, 2014 (840 h)
Only ALT^**X**^	T. 6–1	T. 7–1	T. 8–1	T. 9–1	T. 10–1	T. 11–1
ALT + 300 mg/L HA^W^	T. 6–2	T. 7–2	T. 8–2	T. 9–2	T. 10–2	T. 11–2
ALT + 600 mg/L HA	T. 6–3	T. 7–3	T. 8–3	T. 9–3	T. 10–3	T. 11–3
ALT + 900 mg/L HA	T. 6–4	T. 7–4	T. 8–4	T. 9–4	T. 10–4	T. 11–4

^**Z**^The date of transferring into glasshouse.

^**Y**^Chilling hour, which has been interpreted in the table footnotes of Table 3.

^**X**^ALT: **a**rtificial **l**ow **t**emperature.

^**W**^HA: **h**umic **a**cid.

### Morphological observations and data analysis

After transferring the potted crowns of “T. 1-*” to “T. 11-*” into the glasshouse (Tables 3 and 4), sprouting, stem elongation, growth and flowering were all recorded to evaluate the effects of forcing culture under two types of chilling treatment and three exogenous reagent treatments. The meaning of these morphological indices and interpretation of their corresponding abbreviations were presented in S1, S2, S3 and S4 Tables. The experiments mentioned previously were all performed in a completely randomized design, and Analysis of Variance (ANOVA) was used to determine the statistical significance of the differences using SPSS 16.0.

The research framework is summarized in Fig 2 for a clear understanding of the entire study.

## Results and analyses

### Observation and comparison of the annual growth cycles of three representative cultivars

During the years 2012 to 2017, ‘Hang Baishao’ showed clear early-flowering in Hangzhou, while ‘Jinzan Ciyu’ had an intermediate flowering period and the performance of ‘Taohua Feixue’ was typical of late-flowering cultivars (Fig 3). ‘Hang Baishao’ had the shortest dormancy and earliest sprouting period, the longest flowering duration and green-leaf stage, and the latest senescence period. These phenological traits observed from five to six consecutive years could indicate that this native peony has a significantly longer ornamental period and probably better adaptability than the other two northern cultivars in the relatively warmer climate of Hangzhou.

**Fig 4:**
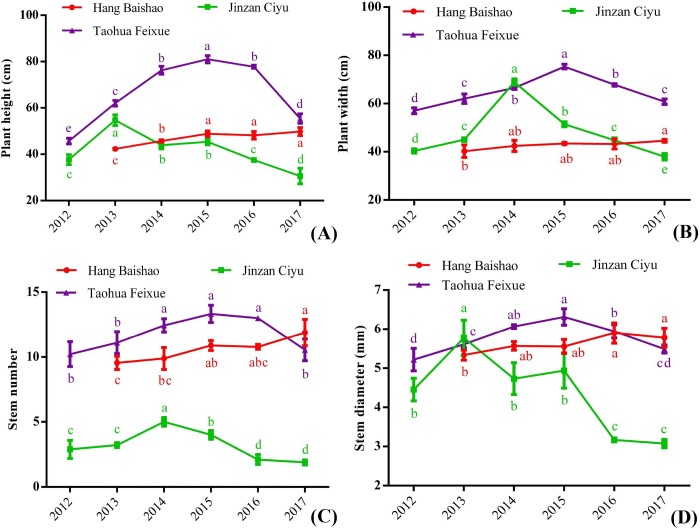
Observation and comparison of the growth performance of three cultivars over six years. The error bars represent the standard deviation, and significance levels are 0.05 in all figures and tables of this study. ‘Hang Baishao’ crowns were introduced and planted in autumn of 2012 for the first time, so all the data related to ‘Hang Baishao’ were recorded from 2013 onward. These will not be repeated in the footnotes of the following figures and tables.

**Fig 5:**
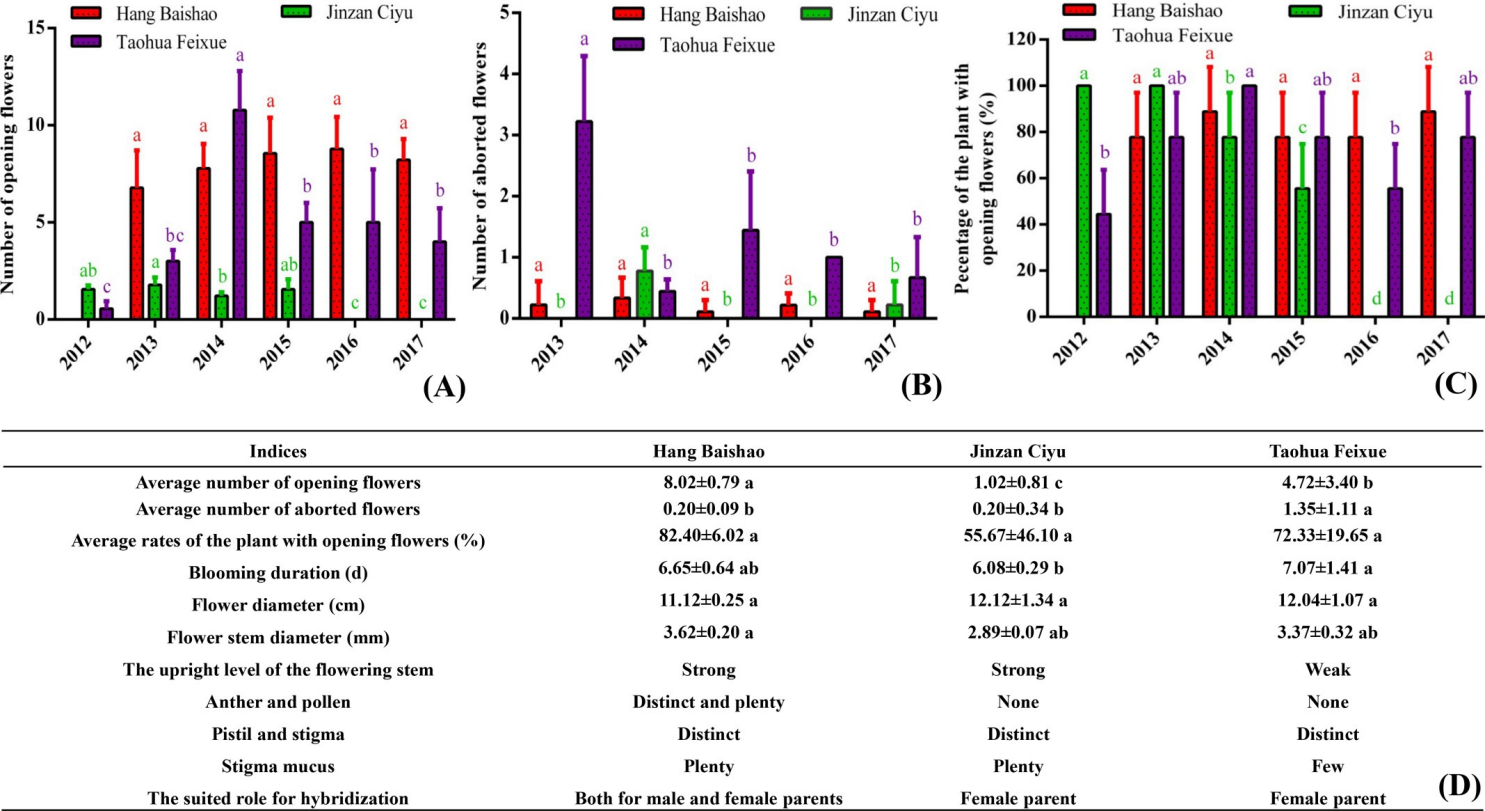
Observation and comparison of the flowering performance of three cultivars over six years. Differences were compared among different years for each cultivar in Fig 5A, 5B and 5C and compared among different cultivars for each index in Fig 5D. Values represent the averages from three replicates ± standard deviation.

Figs 4 and 5 show the growth and flowering of three cultivars from 2012 to 2017. Some important indices, such as plant height, stem number (Fig 4) and number of open flowers (Fig 5A) of ‘Hang Baishao’, increased steadily after introduction in five consecutive years. However, ‘Jinzan Ciyu’ and ‘Taohua Feixue’ gradually grew weaker. Most indices decreased significantly after two to four years (Figs 4 and 5). The average numbers of open flowers of ‘Jinzan Ciyu’ and ‘Taohua Feixue’ were clearly fewer than those of ‘Hang Baishao’ (Fig 5D). ‘Jinzan Ciyu’ had no flowers beginning in 2016 (Fig 5A), while flowering of ‘Taohua Feixue’ was relatively stable for four years after introduction (Fig 5A and 5C).

### Pollen viability and hybridization

#### Exploring the appropriate period and optimal time for observing pollen germination and the necessity of CaCl_2_ application for pollen culture

**Fig 6:**
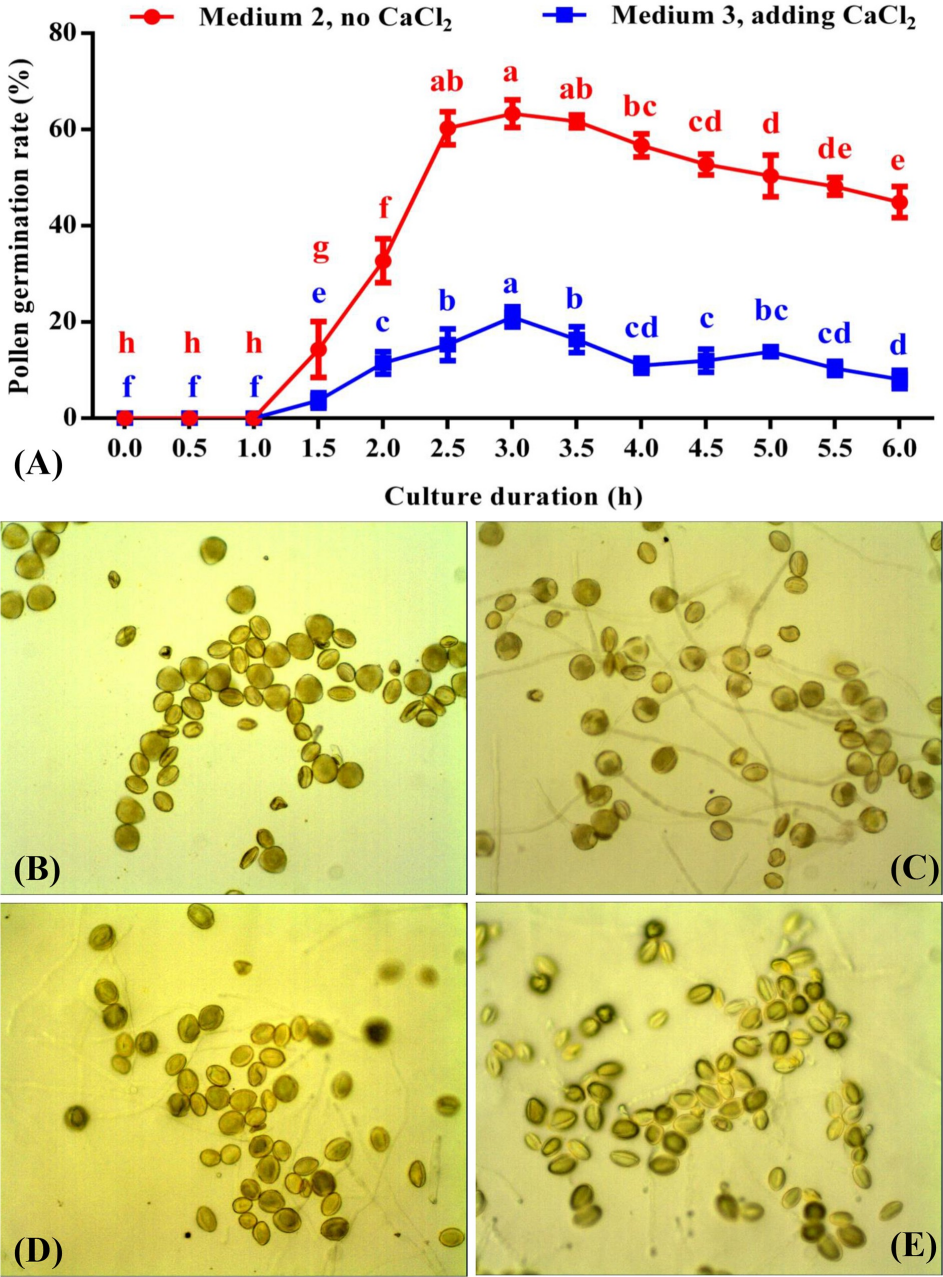
Observation of pollen germination of ‘Hang Baishao’ with the extension of culture duration. (A): comparison of pollen germination rates between media 2 and 3 (Table 2); (B) germination and tube elongation of pollens cultured on medium 2 (Table 2, no CaCl_2_) for 1.0 h; (C): 3.0 h; (D): 5.0 h; (E): 6.0 h.

The germination rates increased sharply between 1.0 h and 2.5 h in both media (Fig 6A, 6B and 6C) but then gradually decreased thereafter (Fig 6A, 6D and 6E). The appropriate period for measuring the pollen germination rate of ‘Hang Baishao’ was between 2.5 and 3.5 h because the tubes were distinct, and germination rates were high and stable during this period (Fig 6). The optimal observation time appears to be 3.0 h after inoculation because the germination rates in media 2 and 3 both reached maximal levels, and pollens and tubes were also clearest in this time (Fig 6A and 6C). Additionally, adding 25 mg/L CaCl_2_ significantly inhibited pollen germination (Fig 6A, media 2 and 3 in Table 2).

#### Determining the agar concentration in medium to reduce pollen submergence

Four types of medium were prepared to optimize the pollen culture of ‘Hang Baishao’ (Table 2). All pollen germination rates were recorded at 3.0 h after inoculation (the optimal observation time) based on previous results (Fig 6). The germination rate of medium 1 was obviously lower than those of media 2 and 4 (Table 2). The difference in germination rates between media 2 and 4 was not significant; therefore, either of these two media is suitable for the pollen culture of ‘Hang Baishao’. However, medium 2 is more economical because it requires less agar. Finally, we selected the value “63.30 ± 0.77” obtained from medium 2 as the final pollen germination rate based on the “GS method” (Tables 2 and 5).

**Table 5:** Pollen viabilities of ‘Hang Baishao’ based on the different methods.

	GS method	TTC method	IKS method	ACS method
Pollen viability (%)	63.30±0.77 a^Z^	46.32±2.42 d	53.85±2.12 c	58.91±1.09 b

^Z^The meanings of symbols a, b, c and d in this table are the same with those in Table 2.

**Fig 7:**
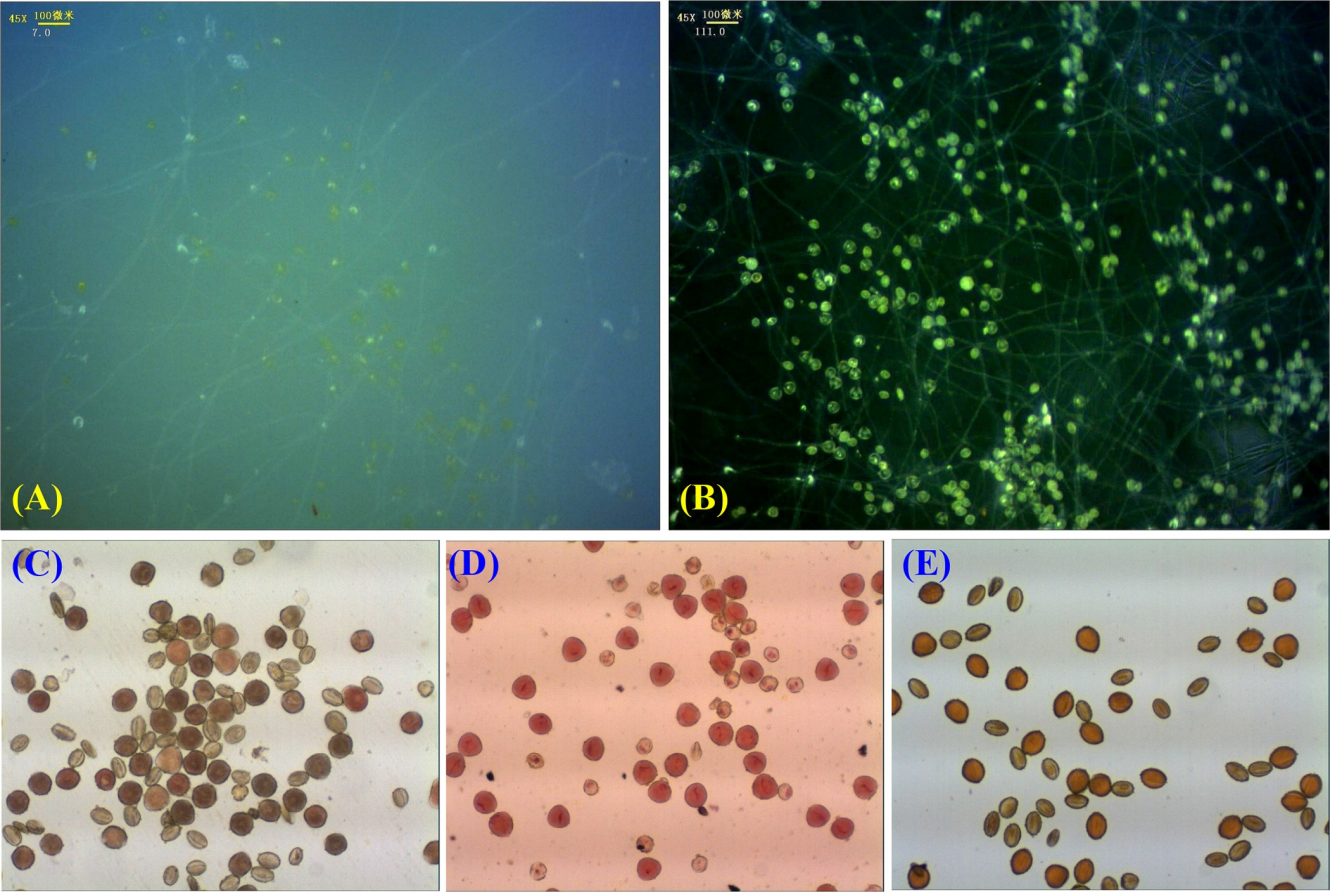
Observation of pollen germination based on different staining methods. (A): germination method using liquid culture medium without black background; (B): germination method using liquid culture medium with black background; (C): the TTC staining method; (D): the ACS method; (E) the IKS staining method.

In addition, we tried to culture the pollen grains in liquid media in glass dishes, and their tubes could germinate and elongate visibly (Fig 7A and 7B). However, the calculations of germination rate were very difficult due to the limitations in lens visualization; the tangled tubes and easily floating pollen grains in the media complicated the process.

#### Measurement of pollen viability of ‘Hang Baishao’ using staining methods

Viability staining of pollen has been proven to be a convenient and effective method, although the pollen viability obtained from staining methods is often lower than those from germination methods (Fig 7C, 7D and 7E). According to Table 5, pollen viabilities of ‘Hang Baishao’ were different acquiring from three staining methods (TTC, ACS and IKS). The ACS method had the relatively closest value to that measured by the germination method. Overall, with over 50% viability, we believe that ‘Hang Baishao’ pollen collected and stored as described is adequate for pollination and hybridization. Stamens of ‘Jinzan Ciyu’ and ‘Taohua Feixue’ were all petaloid, so we could not collect their pollen to do reciprocal crosses with ‘Hang Baishao’.

#### Morphological observations of F1 offspring

**Fig 8:**
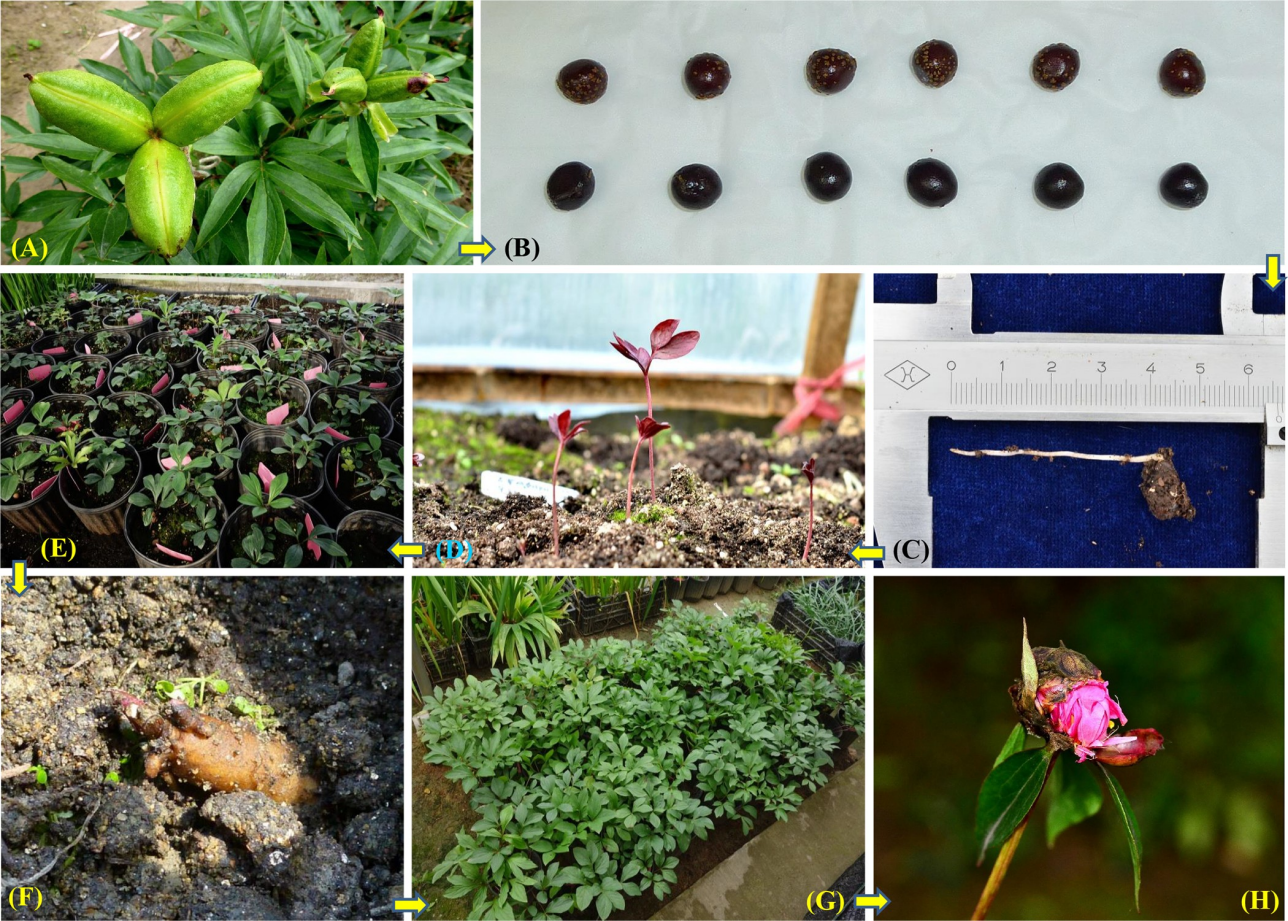
The important seasonal phases of hybridization and cultivation from 2013 to 2017. (A) significantly different sizes between hybrid (left) and self-pollinated (right) follicles in May 2013; (B): hybrid seeds collected in July 2013; (C): rooting of hybrid seeds in November 2013; (D): germination of hybrid seedlings in February to March 2014; (E): one-year-old seedlings grew well in May 2014; (F): tender crowns emerged in September 2014; (G): two-year-old hybrid seedlings grew vigorously in May 2015 (H): flower buds emerged after four years but were all aborted in April 2017.

Hybrid follicles were visibly larger than self-pollinated follicles (Fig 8A). Hybrid seeds were collected and sown immediately in early or mid-July (Fig 8B) and rooted after four months (Fig 8C). One-year seedlings were germinated in the following February to March (Fig 8D), and average heights of seedlings reached a maximum in June at 7.70 cm (‘Hang Baishao’ × ‘Hang Baishao’), 7.83 cm (‘Hang Baishao’ × ‘Jinzan Ciyu’) and 8.60 cm (‘Hang Baishao’ × ‘Taohua Feixue’) (Fig 8E, Table 6).

**Table 6:** Statistics of the hybridization among the three cultivars from 2013 to 2016.

	MP^Z^: Hang Baishao; FP^Y^: Hang Baishao	MP: Hang Baishao; FP: Jinzan Ciyu	MP: Hang Baishao; FP: Taohua Feixue
Seed bearing years	2013, 2014, 2015, 2016	2013, 2014, 2015, 2016	2014, 2016
Seed number per follicle	3.95±0.27 b^X^	5.80±0.27 a	0.35±0.14 c
Average weight of one seed (g)	0.32±0.01 a	0.34±0.02 a	0.13±0.07 b
Seed diameter paralleled with ventral suture (mm)	8.30±0.09 c	9.55±0.21 b	10.63±0.37 a
Seed diameter vertical with ventral suture (mm)	6.40±0.14 b	6.34±0.09 b	7.01±0.13 a
Seed germination rate (%)	92.00±4.47 a	53.00±8.37 b	10.00±22.36 c
Plant height of one-year seedling (cm)	7.70±0.08^**W**^	7.83±0.20	8.60±0.00
Plant height of two-year seedling (cm)	27.68±0.63	29.65±0.85	32.40±0.00
Years needed for generating flower buds	3.00±0.00	3.00±0.00	—
Plant height in the year that generated flower buds (cm)	45.01±1.52	47.45±1.12	—
Percentage of aborted flower (%)	6.00±5.48	100.00±0.00	—

^**Z**^MP: male parent

^**Y**^FP: female parent

^X^The meanings of symbols a, b, c and d in this table are the same with those in Table 2

^**W**^We only acquired one F1 seedling of the combination between ‘Hang Baishao’ and ‘Taohua Feixue’, so we could not obtain the significance of difference for the last five indices of this table.

Tender crowns emerged in September of the second year, which was a crucial phase for the subsequent growth and development of hybrid plants (Fig 8F). Two-year-old plants grew much stronger and taller than one-year-old plants (Fig 8G, Table 6). Hybrid plants of the cross between ‘Hang Baishao’ and ‘Jinzan Ciyu’ generated flower buds after four years. However, all the flower buds were aborted in the spring of 2017 (Fig 8H, percentage of aborted flowers shown in Table 6). We have not yet acquired flower buds of hybrid plants from the cross between ‘Hang Baishao’ and ‘Taohua Feixue’.

### Forcing culture of ‘Hang Baishao’ plants in Treatment Ⅰ

**Fig 9:**
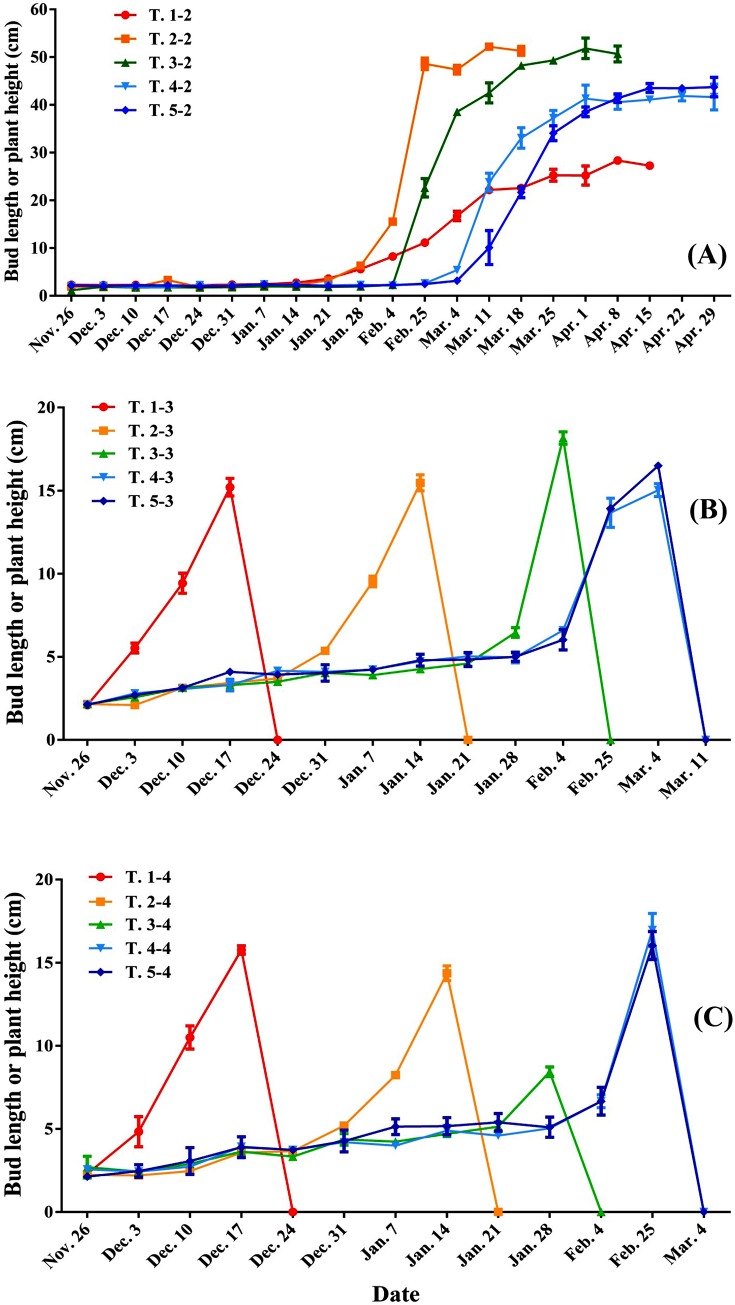
Sprouting and growth of potted ‘Hang Baishao’ plants in Experiment I. (A): natural low temperature + 5-azacytidine; (B): natural low temperature + GA_3_; (C): natural low temperature + 5-azacytidine + GA_3_.

**Fig 10:**
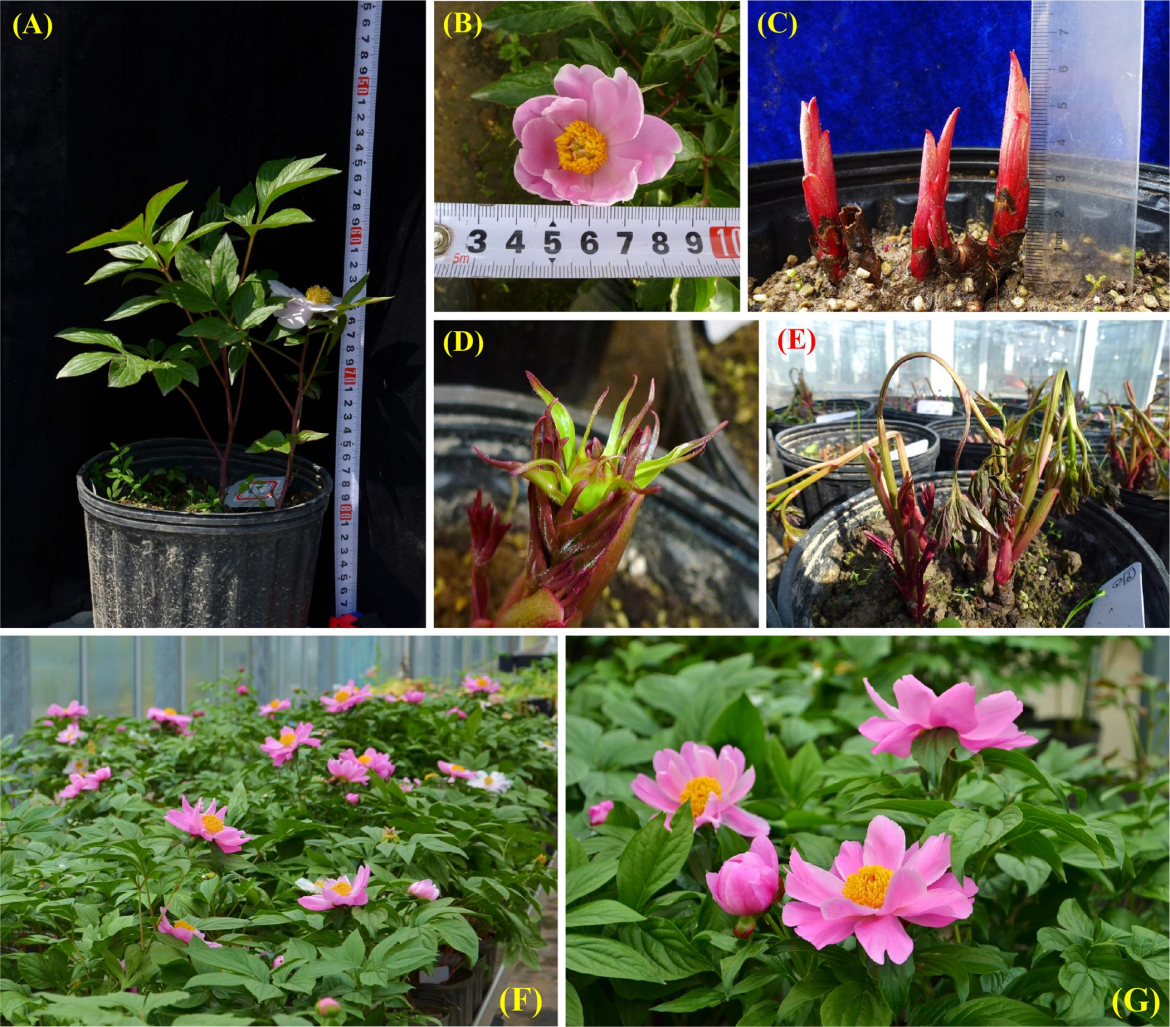
Sprouting, growth and flowering of potted ‘Hang Baishao’ in glasshouse. (A)-(B): the plants of ‘Hang Baishao’ accepted short chilling duration and painted by 5-azacytidine, then they were partially dwarfed and generated small and abnormal flowers; (C)-(D): immediate sprouting and early-visible flower buds of the “T. *-3” and “T. *-4” plants that were irrigated by GA_3_; (E): fallen and withered plants of “T. *-3” and “T. *-4”; (F): the flowering landscape of ‘Hang Baishao’ treated by HA and artificial chilling in the group “T. 10-*” in mid-February; (G): highly ornamental properties of one potted plant in mid-February in the subgroup “T. 10–2” treated by four weeks of chilling and 300 mg/L HA, which had clear commercial value as potted or cut-flower for the market of the Chinese Spring or Lantern Festival.

Fig 9A shows the sprouting and plant growth of ‘Hang Baishao’ following natural chilling and 5-azacytidine treatments. The potted plants grew better with increasing chilling accumulation. Based on the data of DEA (full name and meaning of this index abbreviation was shown in the footnotes of Table 7) in Table 7 and the data of BBP and FBP in S1 Table (full version of Table 7), the treatment of “natural chilling + 5-azacytidine” forced the sprouting of T. 2–2 plants and increased their APW compared to T. 1–2 with chilling treatment only (previously published data)[1]. It is interesting to find that a few plants painted with 5-azacytidine in T. 1–2 and T. 2–2 became dwarfs and their flower diameters were also clearly small (Fig 10A and 10B).

**Table 7:** Observation of the bud break and growth of ‘Hang Baishao’ after transfer into a glasshouse with natural chilling and 5-azacytidine treatments.

Treatment	DFS^Z^ (d)	DEA^Y^ (d)	ANS^X^	APH^W^ (cm)	APW^V^ (cm)
T. 1–2^**U**^	40.00 ± 2.00 a^**T**^	87.67 ± 3.21 a	3.14 ± 0.29 b	27.29 ± 0.46 c	25.23 ± 0.58 c
T. 2–2	24.33 ± 2.08 b	41.00 ± 1.00 b	3.00 ± 0.14 b	51.31 ± 0.94 a	40.32 ± 1.36 a
T. 3–2	11.00 ± 1.00 c	18.33 ± 0.58 c	2.95 ± 0.16 b	50.68 ± 1.66 a	35.27 ± 1.78 b
T. 4–2	—^**S**^	10.00 ± 2.00 d	3.29 ± 0.29 ab	41.64 ± 2.71 b	35.77 ± 1.74 b
T. 5–2	—^**R**^	—^**R**^	3.62 ± 0.22 a	43.72 ± 2.05 b	38.02 ± 1.66 ab

^**Z**^**DFS**: average number of Days between the transfer-date and date of the First plant Sprouting in greenhouse

^**Y**^**DEA**: average number of Days between the transfer-date and date of stem Elongated visibly for All plants

^**X**^**ANS**: Average Number of mature and normal Stems per plant during the full flowering period in the year 2013

^**W**^**APH**: Average Plant Height per plant during the full flowering period

^**V**^**APW**: Average Plant Width per plant during the full flowering period

^**U**^The data of T. 1–1, T. 2–1, T. 3–1, T. 4–1 and T. 5–1 are no longer listed in Tables 7 and 8[1]. The reason has been elaborated in the footnotes 3 of S1 and S2 Tables.

^**T**^The symbols of a, b, c and d in this table indicate the statistical significancess of the differences. For the Tables 7 to 10 and S1, S2, S3 and S4 Tables associated with the forcing culture, significances of differences were all compared within each one column of this table, and standard deviations were added after average values (significance levels = 0.05)

^**S**^The first bud sprouting and stem elongation of T. 4–2 were visible before those being transferred into glasshouse, so the relevant data are inexistent

^**R**^The plants of T. 5–2 were not transferred into glasshouse, so values of DFS and DEA are also inexistent, and values of other T. 5–2 indices in Tables 7 and 8 were observed outside of the glasshouse.

The plants of “T. *-3” and “T. *-4” sprouted and grew rapidly after GA_3_ irrigation (Fig 10C), and many flower buds emerged immediately when the mixed bud scale dehisced (Fig 10D). However, all plants drooped and withered when the stem height reached approximately 15.00 to 18.00 cm (Fig 10E). Thus, we defined all the plant heights after stem withering as 0.00 cm in Fig 9B and 9C.

The effect of 5-azacytidine was not obvious in the accelerating flowering period of ‘Hang Baishao’. The differences of ANO, BDF, AOD, NOF and POF values between the “T. 1-*” (published data)[1] and “T. 2-*” plants were irregular (Table 8 and S2 Table). 5-azacytidine and chilling could not obviously increase the flower number, flowering duration and advance the flowering compared to plants that only received the chilling treatments[1].

**Table 8:** Flowering performance of ‘Hang Baishao’ after transfer to a glasshouse with natural chilling and/or 5-azacytidine treatments.

Treatment	BDF^Z^	NPO^Y^	ANO^X^	ANA^W^	AOD^V^ (d)
T. 1–2	Apr. 08	1.00 ± 0.00 c^U^	0.09 ± 0.08 c	0.19 ± 0.09 c	7.50 ± 0.71 ab
T. 2–2	Mar. 06	3.00 ± 0.00 b	0.86 ± 0.15 b	0.29 ± 0.00 bc	8.09 ± 0.47 a
T. 3–2	Mar. 26	6.33 ± 0.58 a	3.19 ± 0.09 a	0.48 ± 0.08 b	7.93 ± 0.27 a
T. 4–2	Apr. 18	7.00 ± 0.00 a	3.19 ± 0.17 a	1.14 ± 0.25 a	6.85 ± 0.06 bc
T. 5–2	Apr. 19	6.67 ± 0.58 a	3.33 ± 0.17 a	1.09 ± 0.08 a	6.27 ± 0.20 c

^**Z**^**BDF**: the Beginning Date of the first Flower opening

^**Y**^**NPO**: average Number of Plants with Opening flower per replicate

^**X**^**ANO**: Average Number of Opening flowers per plant (NOF/7)

^**W**^**ANA**: Average Number of Aborted flowers per plant (NAF/7)

^**V**^**AOD**: Average Opening Days per flower

^U^The meanings of symbols a, b and c in this Table have been described in the footnote of Table 7.

### Forcing culture of the ‘Hang Baishao’ plants under Treatment Ⅱ

Artificial chilling treatments clearly promoted sprouting and flowering (Tables 9 and 10, S3 and S4 Tables). When chilling accumulated as the transfer date was delayed, the values of FBP, ANS, ADS, APH, APW (Table 9 and S3 Table), NPO, PPO, NOF, ANO, NAF, ANA and PAF (Tables 10 and S4 Table) generally increased, while DFS (Table 9), DFF and POF (Tables 10 and S4 Table) decreased in the six treatment groups. The values of ALL, ALW, ALT, RCC (S3 Table), AOD and ADF (S4 Table) changed erratically.

**Table 9:** Bud break and plant growth of ‘Hang Baishao’ after transfer to a glasshouse with artificial chilling and HA treatments.

Treat-ment	DFS^Z^ (d)	ANS^Y^	ADS^X^ (cm)	APH^W^ (cm)	APW^V^ (cm)
T. 6–2 ^U^	28.67 ± 1.15 b^T^	1.00 ± 0.00 i	2.64 ± 0.72 e	18.40 ± 6.38 e	16.37 ± 6.61 f
T. 6–3	28.67 ± 0.58 b	1.33 ± 0.29 i	3.25 ± 0.37 de	16.60 ± 7.27 e	13.80 ± 6.39 f
T. 6–4	33.00 ± 1.00 a	1.33 ± 0.34 i	3.32 ± 1.49 cde	26.98 ± 5.72 d	24.97 ± 3.57 e
T. 7–2	21.67 ± 1.15 d	3.67 ± 0.88 efg	3.35 ± 0.39 cde	31.11 ± 3.76 cd	34.13 ± 1.39 d
T. 7–3	25.00 ± 0.00 c	3.05 ± 0.48 gh	3.93 ± 0.31 abcd	32.32 ± 9.13 cd	27.94 ± 5.50 e
T. 7–4	21.67 ± 0.58 d	2.67 ± 0.34 h	3.83 ± 0.84 abcd	30.84 ± 4.52 cd	35.30 ± 3.16 cd
T. 8–2	14.00 ± 0.00 f	4.00 ± 0.88 def	3.56 ± 0.39 bcde	34.66 ± 1.32 bcd	36.51 ± 3.08 bcd
T. 8–3	14.67 ± 0.58 f	3.34 ± 0.58 fgh	4.79 ± 0.21 a	39.60 ± 3.13 abc	35.87 ± 2.86 cd
T. 8–4	14.00 ± 0.00 f	4.89 ± 0.70 bcd	4.30 ± 0.57 abcd	36.40 ± 2.76 bc	36.67 ± 1.12 bcd
T. 9–2	11.67 ± 1.15 g	4.56 ± 0.20 cde	4.41 ± 0.37 abc	41.43 ± 5.70 ab	35.41 ± 1.48 cd
T. 9–3	18.00 ± 0.00 e	5.00 ± 0.33 bc	4.49 ± 0.47 ab	41.41 ± 4.52 ab	36.24 ± 5.07 bcd
T. 9–4	14.67 ± 0.58 f	6.34 ± 0.58 a	4.23 ± 0.08 abcd	34.55 ± 3.05 bcd	40.10 ± 1.06 abcd
T. 10–2	11.33 ± 0.58 g	4.22 ± 0.51 cdef	4.89 ± 0.67 a	47.40 ± 5.59 a	45.04 ± 0.91 a
T. 10–3	11.33 ± 0.58 g	5.56 ± 0.51 ab	4.53 ± 0.30 ab	42.47 ± 4.25 ab	41.60 ± 0.46 abc
T. 10–4	11.00 ± 0.00 g	4.55 ± 0.39 cde	3.95 ± 0.16 abcd	34.47 ± 0.26 bcd	37.54 ± 4.15 bcd
T. 11–2	14.00 ± 0.00 f	5.78 ± 0.51 ab	4.37 ± 0.49 abc	37.76 ± 1.03 bc	38.76 ± 3.35 abcd
T. 11–3	14.67 ± 0.58 f	5.11 ± 0.38 bc	4.40 ± 0.31 abc	39.50 ± 1.32 abc	40.23 ± 1.82 abcd
T. 11–4	14.00 ± 0.00 f	5.11 ± 0.19 bc	4.26 ± 0.20 abcd	36.59 ± 2.17 bc	42.98 ± 3.57 ab

^**Z**^**DFS**: average number of Days per replicate between the transfer-date and date of the First plant Sprouting

^**Y**^**ANS**: Average Number of mature and normal Stems per plant during the full flowering period

^**X**^**ADS**: Average Diameter of mature and normal Stems per plant during the full flowering period

^**W**^**APH**: Average Plant Height per plant during the full flowering period

^**V**^**APW**: Average Plant Width per plant during the full flowering period

^**U**^The data of T. 6–1, T. 7–1, T. 8–1, T. 9–1, T. 10–1 and T. 11–1 are no longer listed in Tables 9 and 10[1]. The reason has been elaborated in the footnotes 3 of S3 and S4 Tables

^T^The meanings of symbols a, b and c etc. in this Table have been described in the footnote of Table 7.

**Table 10:** Flowering performance of ‘Hang Baishao’ after transfer to a glasshouse with artificial chilling and HA treatments.

Treatment	BDF^Z^	NPO^Y^	ANO^X^	ANA^W^	AOD^V^ (d)
T. 6–2	-^U^	0.00 ± 0.00 g^T^	0.00 ± 0.00 h	0.00 ± 0.00 b	-^U^
T. 6–3	-^U^	0.00 ± 0.00 g	0.00 ± 0.00 h	0.00 ± 0.00 b	-^U^
T. 6–4	Feb. 15	0.67 ± 0.58 fg	0.22 ± 0.19 h	0.00 ± 0.00 b	6.00 ± 0.00 e
T. 7–2	Feb. 6	1.67 ± 0.58 cde	1.11 ± 0.38 efg	0.00 ± 0.00 b	9.50 ± 2.22 bc
T. 7–3	Feb. 6	2.00 ± 0.00 bcd	1.45 ± 0.39 defg	0.22 ± 0.39 ab	8.04 ± 0.56 cd
T. 7–4	Feb. 5	1.67 ± 0.58 cde	0.89 ± 0.19 g	0.11 ± 0.19 ab	11.17 ± 1.26 ab
T. 8–2	Feb. 1	1.33 ± 0.58 def	1.11 ± 0.19 efg	0.11 ± 0.19 ab	7.00 ± 0.33 de
T. 8–3	Jan. 29	2.33 ± 0.58 abc	1.67 ± 0.34 cdef	0.22 ± 0.39 ab	7.13 ± 1.80 de
T. 8–4	Jan. 29	1.67 ± 0.58 cde	1.67 ± 0.34 cdef	0.00 ± 0.00 b	7.33 ± 0.58 de
T. 9–2	Feb. 5	2.00 ± 0.00 bcd	2.22 ± 0.19 c	0.11 ± 0.19 ab	12.03 ± 0.65 a
T. 9–3	Feb. 5	2.00 ± 1.00 bcd	1.55 ± 0.39 cdefg	0.33 ± 0.34 ab	8.31 ± 0.34 cd
T. 9–4	Feb. 8	2.33 ± 0.58 abc	1.78 ± 0.39 cde	0.56 ± 0.20 a	7.92 ± 0.58 cd
T. 10–2	Feb. 16	3.00 ± 0.00 a	4.44 ± 0.20 a	0.11 ± 0.19 ab	10.57 ± 0.62 ab
T. 10–3	Feb. 19	2.67 ± 0.58 ab	3.56 ± 0.51 b	0.22 ± 0.19 ab	9.61 ± 0.54 bc
T. 10–4	Feb. 18	1.00 ± 0.00 ef	1.89 ± 0.19 cd	0.22 ± 0.19 ab	9.69 ± 0.30 bc
T. 11–2	Feb. 28	1.67 ± 0.58 cde	1.22 ± 0.19 defg	0.44 ± 0.20 ab	8.64 ± 1.44 cd
T. 11–3	Feb. 27	3.00 ± 0.00 a	1.78 ± 0.19 cde	0.33 ± 0.34 ab	7.98 ± 0.31 cd
T. 11–4	Feb. 28	0.67 ± 0.58 fg	1.00 ± 1.00 fg	0.56 ± 0.51 a	8.00 ± 0.47 cd

^**Z**^**BDF**: the Beginning Date of the first Flower opening

^**Y**^NPO: Average Number of Plants per replicate with Opening flower

^**X**^**ANO**: Average Number of Opening flowers per plant

^**W**^**ANA**: Average Number of Aborted flowers per plant

^**V**^**AOD**: Average Opening Days per flower

^**U**^All plants of the Tre. 6–2 and 6–3 never bloomed or have aborted flower, so the partial data were unable to be collected

^T^The meanings of symbols a, b and c etc. in this Table have been described in the footnote of Table 7.

The flowering time of ‘Hang Baishao’ was not advanced visibly by means of natural chilling, 5-azacytidine and/or GA_3_ solution treatments (Figs 9 and 10A**–**10E, Tables 9 and 10), whereas flowering was enhanced significantly following combined treatments of artificial chilling and humic acid (Table 10). Among the six groups, “T. 10-*” plants had the maximum average values of FBP, ADS, APH, APW, NPO, PPO, NOF and ANO, the higher values of ANS, the minimum value of DFS and the lower average value of DFF. These are all important indices for evaluating the effects of forcing culture. Therefore, the growth and flowering of group “T. 10-*” were the best compared with those of the other five groups.

T. 10–2 plants bloomed on Feb. 16 (Table 10); their flowering was advanced by more than two months compared to the natural flowering time of ‘Hang Baishao’. This would greatly increase the commercial value of potted ‘Hang Baishao’ in China because mid-February is often the period of the Chinese Spring Festival and Lantern Festival, which are the two most important traditional festivals in Chinese life and culture (Fig 10F and 10G). Additionally, T. 10–2 plants had 4.44 flowers per plant (ANO) and PPO of T. 10–2 was 100% per replicate, which are both the highest values among all treatments (Table 10 and S4 Table). Therefore, artificial chilling treatment in 0–4°C refrigerated storage for four weeks and pot-irrigation with 300 mg/L HA (low concentration) is the best combination for reducing the time to flowering while simultaneously ensuring good flowering quality in ‘Hang Baishao’.

The plants of “T. 11-*” with five weeks of chilling seemed to acquire excessive chilling and had relatively worse growth and flowering than those of the group “T. 10-*”. For example, the average ANO value of the “T. 11-*” plants (1.33 in S4 Table) was much less than that of the “T. 10-*” plants (3.30 in S4 Table); in addition, the average ANA value of the “T. 11-*” plants (0.44 in S4 Table) was the highest among the six treatment groups. These data indicate that four weeks of chilling duration is optimal for both ensuring earlier flowering and the highest flowering quality.

## Discussion

### The warm winter is a more crucial and troublesome problem than the hot summer for the southward planting of herbaceous peony in the Northern Hemisphere

Warm winters and hot summers are two main problems for the large-scale production and application and exuberant flowering of herbaceous peony in subtropical and tropical regions. Severe heat and sunburn in summer both harm the upper leaves and stems and negatively affect the photosynthetic efficiency and carbohydrate accumulation of peony. High soil temperature in summer also damages the peony rhizome. However, these adverse factors only harm some of the tissues and organs of peony. Using shade nets or advanced spray-cooling systems can greatly reduce the heat injury caused by hot summers, although this maintenance will inevitably require additional expenditures and labor.

High winter temperatures are a much more troublesome problem than high summer temperatures. Chilling accumulation is seriously deficient for most herbaceous peony cultivars under the warm winter climate of the low-latitude regions, which leads to incomplete bud dormancy release, and thus results in the following uneven sprouting, dwarf plants and very limited flowering. Therefore, for the geophytes, a warm winter can restrict all the subsequent growth and developmental stages after dormancy in spring and summer and finally leads to rapid degradation of rhizomes and even death[1,12].

**Fig 11:**
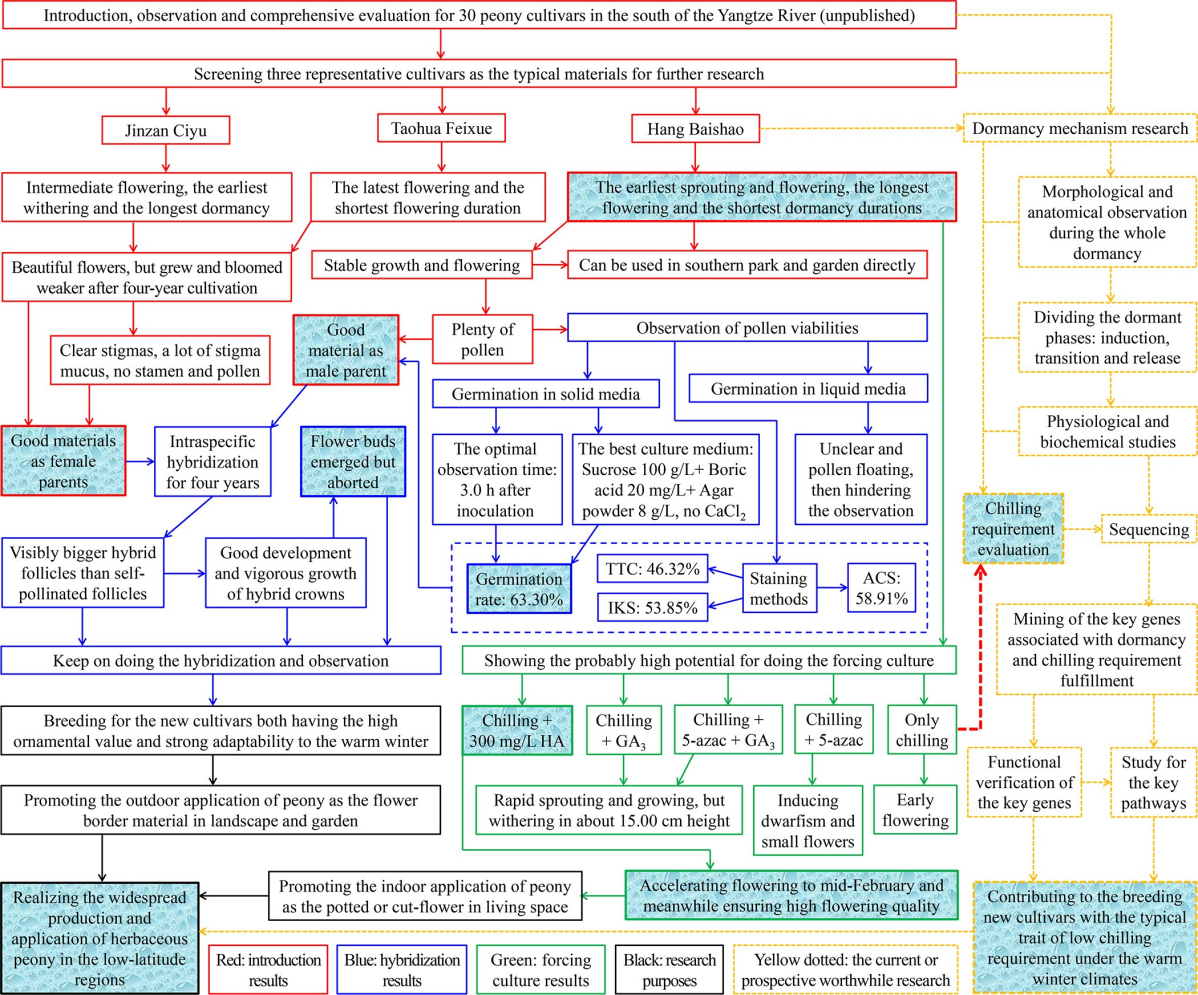
The main results and logistical relationships among different parts of this study.

Reducing these injuries from warm winters is very difficult compared with those from hot summers. It is unrealistic to dig out all peony rhizomes from gardens or parks and transfer them into refrigerated storage or chilling chambers, then transfer them out after enough chilling accumulation, and finally plant them into their original positions again each year. Therefore, breeding new cultivars with low chilling requirements is the only way to solve the warm-winter problem, and studies on chilling requirement traits, bud dormancy release and greenhouse cultivation are certainly indispensable (yellow dotted lines and arrows in Fig 11). Resource introduction is the first step of carrying out these studies[1,12].

### Resource introduction is an indispensable basis for ensuring successful hybridization and reasonable identifying for typical research materials

Intraspecific and interspecific hybridizations play a crucial role in breeding new genotypes with economically desirable traits[23,28,35]. The success of any breeding effort in perennials must be based on the wise screening of male and female parents to reduce potential cross-incompatibility[34]. When developing different cultivars, introducing abundant cultivars and observing and defining their differences in dormancy, sprouting, flowering and senescence are necessary for parent selection and hybridization[14,36,37]. Resource introduction, observation of the annual growth cycle and evaluation of comprehensive adaptability could also provide typical materials with clear differences for further studies on the physiological, biochemical and molecular mechanisms of economically important plants[1,12]. Therefore, painstaking introduction and long-term observation in nursery/field conditions must be made before crossbreeding and undertaking studies on the mechanisms controlling physiology and development.

Taking this study as an example, based on the results of plant introduction and observation of annual growth cycles, the crowns of ‘Jinzan Ciyu’ should be replaced after four to five years of landscape utilization (Fig 5A). ‘Hang Baishao’ and ‘Jinzan Ciyu’ could also be used as good cut-flower materials because of their reduced flower abortion and strong flowering stems (Fig 5B and 5D). In addition, because of its early flowering and prolific pollen production (Fig 3 and Fig 5D), ‘Hang Baishao’ is well suited as a male parent for collecting pollen for future hybridization. The other two cultivars could be used as female parents for hybridization because they are late blooming and lack of anthers and pollen (Fig 3 and Fig 5D). Thus, these results are very helpful for developing hybridization strategy and defining the appropriate application of these three cultivars in landscape and garden.

### The problems of pollen submergence and inhibition of pollen germination by CaCl_2_ are interesting and worthy of attention

Cultivating pollen *in vitro* and then observing germination is frequently used as a time-consuming but dependable method for measuring pollen viability[38]. Here, we highlighted solutions to three problems, which are the determination of the optimal observation time, the necessity of using CaCl_2_, and the problem of pollen loss due to submergence in media which previously inhibited the culture of peony pollen *in vitro*. In this study, most pollen submerged within 3.5 h postinoculation (Fig 6A). In contrast, similar research on pecan found that adding relatively higher agarose concentrations (1.0% instead of 0.5% agarose) could result in reduced pollen growth due to insufficient contact with solid medium[24]. However, for ‘Hang Baishao’ pollen grains, the agar concentration could not be lowered, or the pollen would sink into the media which inhibited pollen tube elongation.

Another peculiar phenomenon is the inhibitory effect of CaCl_2_ on ‘Hang Baishao’ pollen germination, which is also unusual compared with similar studies in other species. The steep tip-to-base gradient of free cytosolic calcium is an essential factor for the generation and elongation of pollen tubes[39]. Exogenous calcium ions could increase this gradient in plant cells and possibly act as a signal to activate pollen germination and/or influence the physical properties of the pollen intine directly[26]. Unexpectedly, CaCl_2_ apparently repressed the germination of ‘Hang Baishao’ pollen in our study, which is worthy of attention.

In addition to these observations, some other preparations should also be made before hybridization, including ensuring stigma acceptability. Seed number per follicle of the combination between ‘Hang Baishao’ and ‘Taohua Feixue’ was markedly lower than that of the combination between ‘Hang Baishao’ and ‘Jinzan Ciyu’ (Table 6). One probable reason is that the stigma mucus of ‘Taohua Feixue’ was scarcely visible (Fig 5D); thus, the stigma acceptability of this cultivar may be very low, and needs to be tested.

### Chilling is crucial for dormancy release and accelerating flowering, but excessive chilling accumulation could inhibit the sprouting of herbaceous peony and induce flower abortion

It is well known that appropriate chilling duration can hasten the flowering of ornamentals[10,12]. Enough chilling accumulation could impact the pathways of carbohydrates, phytohormones or antioxidases and then promote the transition from endodormancy to ecodormancy and subsequent ecodormancy release, sprouting, growth and flowering[7,8,40]. However, excessive chilling could also inhibit flowering and decrease the flower quality of ornamentals, such as *Camellia japonica* L.[41], and peony in this study. The inhibitory effect of excessive chilling on peony flowering was also correlated with faded flower color and increased flower bud abortion[10,13,41]. The average ANA number of the “T. 11-” plants was much higher than that of “T. 10-” plants, which may indicate that too great a duration of chilling reduced the ornamental and commercial value of herbaceous peony (S4 Table).

Flower bud abortion is a frequent phenomenon and very difficult to avoid during forcing culture[10,42], which could possibly result from the limitation of carbohydrates, high temperature, short photoperiod, and abnormal ratio between endogenous hormone and carbohydrate levels[10,43–45]. Excessive chilling accumulation is probably another reason, according to this study. Therefore, avoiding superfluous chilling and determining the optimal chilling requirement for herbaceous peony is a significant and desirable endeavor[1,41]. We have already defined the optimal chilling to be approximately 672.00 hours at 0–4°C in our previous publication[1].

### 5-azacytidine could increase DNA demethylation and inhibit DNA methylation and then induce sprouting and/or flowering

This is the first study to report the impact of 5-azacytidine on sprouting and flowering of herbaceous peony. We found two interesting phenomena: (1) 5-azacytidine could improve sprouting and growth (Table 7 and S1 Table) but not advance flowering for ‘Hang Baishao’ (Table 8 and S2 Table); and (2) the combination of 5-azacytidine and short chilling duration could partly lead to dwarfism and diminished flowers (Fig 10A and 10B). The substitution effect of 5-azacytidine in low temperature and reduction of DNA methylation by 5-azacytidine are widely accepted and are the most likely cause of the observed impact. Chilling accumulation which preconditions vernalization and winter dormancy release, has also been associated with a decrease in DNA methylation and/or an increase in DNA demethylation[46,47]. DNA methylation has been confirmed to gradually decrease with chilling-mediated dormancy, while demethylation has a positive correlation with the release of dormancy [48,49]. 5-azacytidine is a cytosine analogue that can intercalate into DNA[50,51], leading to the inactivation of methylase enzymes, and is known as a “demethylation/demethylating agent” or a “DNA-hypomethylating molecule”[49,50,52–54]. Therefore, 5-azacytidine could partially replace the effect of cold, potentially promoting the progress of DNA demethylation, accelerating the dormancy release and finally inducing earlier sprouting and/or flowering[47,53,55].

5-azacytidine has been experimentally used in seed or cell cultures to improve sprouting and growth and initiate flowering of vegetables and ornamentals, such as *Linum usitatissimum* L.[56], *Chrysanthemum* cultivars[57] and *Pharbitis nil* L.[58]. Our previous study also revealed that spraying 40 mg/L 5-azacytidine solution could rapidly promote the expansion of azalea flower buds, advance their flowering periods from seven to 17 days, and unexpectedly induce many special petaloid stamens[59,60]. For the peonies in this study, we do not recommend 5-azacytidine for promoting dormancy release because of the high price of 5-azacytidine, minimal effect on forcing culture and the resulting plant dwarfism[58]. Even so, the effects of 5-azacytidine on herbaceous peony and azalea are interesting and deserve more attention[59,60].

### GA_3_ can result in the immediate loss of dormancy and sprouting but also induce detrimental growth and development

It is well known that gibberellins play a pivotal role in promoting dormancy release, bud sprouting, stem growth and the network regulation of floral induction pathways[61,62]. After irrigation with GA_3_, the underground buds of ‘Hang Baishao’ sprouted and elongated quickly, and flower buds also emerged early (Fig 10C and 10D), which was similar to results of other studies[63,64]. However, all plants withered (Fig 10E) and the flower buds were all aborted before stem lodging. Similar phenomena were also observed in other peony studies[20,42,65]. Although the rapid internode elongation is possibly the result of increased cell elongation and cell division commonly caused by an inappropriate endogenous hormone ratio (ABA/GA) and excessively high GA_3_ concentrations from the exogenous reagent[20,42,65], the loss of viable growth might result from the imbalance of carbohydrate contents because the rapid shoot elongation is not supported by an equivalent expansion of leaves and subsequent photosynthetic capabilities.

GA_3_ and 5-azacytidine may promote dormancy release synergistically by decreasing DNA methylation. A study on *Nicotiana tabacum* L. reported that application of exogenous GA_3_ could reduce global cytosine DNA methylation so that increased endogenous GA levels in shoot apical tissues during chilling occurred simultaneously and induced flowering. This “antagonistic” relationship may indicate that GA_3_ inhibits DNA methylation by increasing the mobility and/or modification of linker histones[66]. This interesting observation might mean that the mechanisms regarding dormancy release by GA_3_ and 5-azacytidine are similar and might explain why, at least in this system of *N*. *tabacum* L, both induce dormancy release, bud sprouting and/or early flowering[66].

### Relatively low concentrations of humic acid and moderate chilling accumulation could advance the flowering and improve flowering quality of herbaceous peony

It was unexpected that the combination of chilling and humic acid (HA) could enhance flowering quality better than combinations of chilling, 5-azacytidine and/or GA_3_. HA is a famous natural compound derived from the decay and accumulation of organic matter in soil, lignin and peat and is often used in sustainable agriculture[67]. It is known to release soil nutrients and chelate micronutrients and thus make them available for crops. Thus, HA can promote growth and development and increase the yield and stress resistance of economic crops[68,69].

We have abundant experience using HA to improve the flowering and tissue culture of ornamentals[68,70–73]. Our previous work indicated that the harvested flower number per plant of *Gerbera jamesonii* Bolus ex was increased after treatment with 500 mg/L HA, and vase life was extended from 2.00 to 3.66 days under higher HA levels. These positive postharvest performances were most likely derived from calcium accumulation in stems and the hormone-like activity of HA[70]. In another study on flowering in *Lilium* ‘Sorbonne’, we noted that plants bloomed eight days earlier after the treatment of 500 mg/L HA[71,74,75]. The size of tissue cultured ‘Sorbonne’ bulblets could also be increased by a relatively low dose of HA that acted via the positive GA and antioxidant responses[72,73]. Additionally, in the study of *Rhododendron* ‘Zi Hudie’, we found that HA could improve rooting in tissue cultured seedlings and increase endogenous hormone levels and antioxidase activities[68].

The current study is the first report to use HA in forcing culture for herbaceous peony. A low concentration of HA improved bud sprouting and growth, hastened flowering and simultaneously ensured greater flowering quality[72,73,76]. The moderate application of HA may increase the efficiency of crowns for absorption of soil nutrient elements, promote organic compound accumulation, change the endogenous hormone balance, and improve crown enrichment and expansion, bud sprouting, plant growth and flowering[68,70,71]. However, excessive application of HA might lead to marked flower abortion (high value of NAF, ANA, PAF in S4 Table) and inhibit the growth and flowering of ‘Hang Baishao’, as we observed in our previous study on *G*. *jamesonii*[70]. In summary, chilling in 0–4°C refrigerated storage for four weeks and pot-irrigation with 300 mg/L HA is the optimal combination for forcing flowering and ensuring the flowering quality of ‘Hang Baishao’.

### Expanding the applications of herbaceous peony in the low-latitude regions should be carried out indoors and outdoors simultaneously

The purpose of this study is to expand the southern range for planting of herbaceous peony in China and other subtropical or tropical regions of the Northern Hemisphere through “outdoor” work, i.e., resource introduction, annual growth cycle observation and breeding in nursery or field conditions (red and blue frames and arrows in Fig 11), and simultaneously, through “indoor” work, i.e., facilitating peony production and utilization as a potted and cut-flower in glasshouses to reduce shortfalls in the winter market, prolong the flowering duration and collect pollen in advance, for facilitating breeding efforts with late-flowering peonies (green frames and arrows in Fig 11)[10,16,17,20]. Fig 11 distinctly summarizes the main data and results, logistical relationships and purpose of this study. We believe the success of increasing the range of one horticultural crop to other regions with different climate should be based on “outdoor” and “indoor” practices simultaneously.

## Supporting information

S1 Table Observation on the sprouting and growth of *Paeonia lactiflora* ‘Hang Baishao’ with natural chilling and 5-azac treatments after being transferred into glasshouse. (XLSX)

S2 Table Observation on the flowering performance of *Paeonia lactiflora* ‘Hang Baishao’ with natural chilling and 5-azac treatments after being transferred into glasshouse. (XLSX)

S3 Table Observation on the sprouting and growth of *Paeonia lactiflora* ‘Hang Baishao’ with artificial chilling and humic acid treatments after being transferred into glasshouse. (XLSX)

S4 Table Observation on the flowering performance of *Paeonia lactiflora* ‘Hang Baishao’ with artificial chilling and humic acid treatments after being transferred into glasshouse. (XLSX)
